# Factors Moderating the Relationship Between Childhood Trauma and Premorbid Adjustment in First-Episode Schizophrenia

**DOI:** 10.1371/journal.pone.0170178

**Published:** 2017-01-20

**Authors:** S. Kilian, J. K. Burns, S. Seedat, L. Asmal, B. Chiliza, S. Du Plessis, M. R. Olivier, M. Kidd, R. Emsley

**Affiliations:** 1 Department of Psychiatry, Stellenbosch University, Cape Town, South Africa; 2 Department of Psychiatry, University of KwaZulu-Natal, Durban, South Africa; 3 Institute for Health Research, University of Exeter, Exeter, United Kingdom; 4 Centre for Statistical Consultation, Stellenbosch University, Cape Town, South Africa; Johns Hopkins University Bloomberg School of Public Health, UNITED STATES

## Abstract

Childhood trauma is a recognised risk factor for schizophrenia. It has been proposed that childhood trauma interferes with normal neurodevelopment, thereby establishing a biological vulnerability to schizophrenia. Poor premorbid adjustment is frequently a precursor to schizophrenia, and may be a manifestation of neurodevelopmental compromise. We investigated the relationship between childhood trauma and premorbid adjustment in 77 patients with first-episode schizophrenia spectrum disorders. We also investigated possible mediating roles for other selected risk factors in the relationship. We found several significant correlations between different trauma types and both social and academic premorbid adjustment from childhood to late adolescence. There were no significant moderating effects for family history of schizophrenia or family history of psychiatric disorder. History of obstetric complications, substance abuse and poor motor coordination weakened some of the associations between childhood trauma and premorbid adjustment, while poor sequencing of motor acts strengthened the association. Our results confirm previous studies indicating an association between childhood trauma and premorbid adjustment. Results indicate a general rather than specific association, apparent with different types of trauma, and affecting both social and academic components of premorbid adjustment across childhood, early and late adolescence. Further, our results suggest a complex interplay of various risk factors, supporting the notion of different pathways to psychosis.

## Introduction

Schizophrenia is widely regarded as a neurodevelopmental disorder, with genetic and environmental risk factors interacting to contribute to altered brain development and later manifestation of the illness [1]. Premorbid adjustment (i.e. an individual’s functioning across social and academic domains from childhood through to early adulthood prior to the onset of psychosis) has long been a focus of interest in schizophrenia research [2]. Deficits in premorbid adjustment are reported consistently as a precursor to schizophrenia [3], and as such provide supportive evidence for the neurodevelopmental model [4–5]. For example, poor social adjustment during childhood has been proposed as an early manifestation of impairment of developmental trajectories that evolve into deficits in social cognition and later into schizophrenia [6].

Childhood trauma in the form of abuse or neglect has also been linked to schizophrenia, increasing the risk of developing the illness [7], and contributing to poorer treatment outcomes [8]. However, the nature of the relationship between childhood trauma and schizophrenia is not well understood. Evidence from animal and human research suggests that the effects of exposure to childhood trauma may be enduring, and affect behaviour, emotions and cognition into adult life [9]. Whether the effects of childhood trauma are direct, or whether they are mediated or moderated by other risk factors, is not clear. It is well established that people living with psychotic disorders, in comparison to the general population and healthy controls, are more likely to have a history of childhood trauma. It has been hypothesized that developmental trauma lies on the “affective pathway” to psychosis [10–11] and it is evident that a history of childhood trauma has an impact on symptom expression [12] and severity [13], leading to poorer patient outcomes [8]. One possibility is that childhood trauma, through psychological or biological mechanisms, interferes with normal neurodevelopment, thereby establishing a biological vulnerability in a pre-schizophrenic individual. If this is the case, then an association between childhood trauma and premorbid adjustment would be anticipated. Indeed, studies have documented such a relationship in people living with psychosis. In a retrospective chart analysis of 658 broadly defined first-episode psychosis patients, Conus [14] reported that 34% had been exposed to sexual and/or physical abuse during childhood, and that these patients were more likely to have poorer premorbid functioning. Tikka [15] investigated 20 patients at clinical high risk for psychosis and 30 normal control subjects. Childhood trauma scores were higher, and premorbid adjustment poorer, in patients than in controls. Emotional abuse was significantly associated with poor premorbid adjustment in late adolescence and overall trauma was significantly correlated with poor general premorbid adjustment. Stain [16] specifically investigated the association between childhood trauma and social functioning in 233 patients with a first-episode of schizophrenia-spectrum disorder and reported that childhood trauma was significantly associated with poorer premorbid social functioning in childhood, early adolescence and late adolescence. Finally, Alameda [17] investigated the relationship between childhood abuse and functional outcome in 225 early psychosis patients. They found that physical and sexual abuse were associated with poor premorbid social functioning in early adolescence and that the age of first trauma exposure correlated significantly with social functioning in patients at follow-up.

A relationship between childhood trauma and premorbid adjustment has therefore been established. However, the nature of the relationship has not been extensively investigated. It is not known, for example, whether specific types of childhood trauma have a stronger relationship with premorbid adjustment, and whether there is a differential impact on academic and social premorbid adjustment. Another important question is whether childhood trauma acts on its own or whether other risk factors moderate or mediate its effect on premorbid adjustment. Other risk factors include, amongst others, obstetric complications [18], neurological soft signs, substance abuse [18] and a family history of the illness [19].

In this study we investigated the relationship between childhood trauma and premorbid adjustment in patients diagnosed with a first episode of schizophrenia-spectrum disorders (schizophrenia, schizophreniform or schizoaffective disorder). We examined whether specific types of trauma had stronger relationships with premorbid adjustment, and whether academic and social adjustment were differentially affected. Further, we explored a possible moderating role for other selected risk factors in the relationship between childhood trauma and premorbid adjustment. Risk factors considered in this study were a family history of schizophrenia and a family history of psychiatric disorder (as proxies for genetic risk), obstetric complications, neurological soft signs and substance abuse. We hypothesized that: (a) childhood abuse would be associated with poorer premorbid adjustment than childhood neglect; (b) childhood trauma would have a greater negative impact on premorbid social adjustment than on academic adjustment; and (c) other investigated risk factors for psychosis would have a synergistic effect and serve to strengthen the association between childhood trauma and premorbid adjustment (i.e. higher levels of trauma would be associated with poorer premorbid adjustment).

## Materials and Methods

### Subjects and Study Setting

Participants were recruited from first admissions to Tygerberg and Stikland hospitals, and from community clinics in the greater Cape Town area. Inclusion criteria were: men and women; in- or out-patients; aged 16 to 45 years; experiencing a first psychotic episode; and meeting DSM-IV TR (Diagnostic and Statistical Manual of Mental Diseases, Fourth Edition, Text Revisions) diagnostic criteria for schizophrenia, schizophreniform or schizoaffective disorder. Exclusion criteria were: a lifetime exposure to antipsychotic medication for longer than 4 weeks; any serious or unstable general medical condition; substance-induced psychosis; and an educational level of lower than Grade 7. A group of healthy controls, matched for age, gender, ethnicity and educational status was recruited from the same catchment area as the patient group. Controls were excluded if they met the criteria for any past or current psychiatric disorder. The study described in this manuscript is nested within a larger first-episode psychosis study. In the larger study n = 234 patients were approached of which n = 19(8%) declined to participate. In the end we collected data on childhood trauma in n = 77 patients and 52 healthy controls. Permission to conduct the study was obtained from the Health Research Ethics Committee of the Faculty of Medicine and Health Sciences, Stellenbosch University. Written, informed consent was obtained from all participants and in the case of participants younger than 18 years we obtained written, informed consent from parents or guardians.

### Assessments

Patients were assessed with the Structured Clinical Interview for DSM-IV [SCID] [20]. Detailed assessments of demographic, obstetric complications (any complications experienced in the antenatal, perinatal and postnatal (up to 6 weeks post delivery) period), clinical and family history of any serious psychiatric disorders amongst first and second-degree relatives were obtained through interviews with patients and caregivers, according to a structured schedule. Information about substance use was obtained through clinical interviews with patients, collateral information was obtained from family members and urine screening tests were done for cannabis, methamphetamine and methaqualone.

Participants and controls were assessed with the Childhood Trauma Questionnaire (CTQ) short form, which has good construct validity [21]. The instrument has 28 Likert-type items (25 items assessing clinical symptoms and 3 validity items identifying underreporting), 5 subscales (sexual abuse, physical abuse, emotional abuse, physical neglect and emotional neglect), and yields a total score [22]. Subscale scores range from 5–25 and the total scale score (sum of the 5 subscales) ranges from 25–125 [21]. We included subscale and total scores in the analyses. Premorbid functioning was assessed in patients using the Premorbid Adjustment Scale (PAS), which has good validity and reliability (inter-rater reliability estimates ranged from 0.74–0.85 [2]. The PAS assesses levels of functioning in areas of sociality, peer relationships, school performance, adaption to school, and social sexual aspects across four developmental periods: childhood (up to 11 years of age); early adolescence (12–15 years of age); late adolescence (16–18 years of age); and early adulthood (older than 19 years of age). In childhood, social sexual aspects are not assessed. The PAS also includes a general section that assesses highest level of education, changes in work and school performance, and quality of life [2]. Item scores range from 0–6, with higher scores indicative of worse premorbid adjustment. Normally items in each subscale are summed and divided by the sum of the highest possible score for items completed. The total scale score is the average of the subscale scores for all subscales [2]. However, given the evidence that social and academic functioning are distinct components of premorbid functioning [23–24], we compared premorbid functioning according to academic and social domains separately, across the developmental life stages from childhood to late adolescence. We followed the procedure of Allen [24] by excluding data from the adult period, as early manifestations of illness can affect these scores.

We used the Neurological Evaluation Scale (NES) to assess neurological abnormalities [25]. The NES assesses three domains (sequencing of motor acts, motor coordination, and sensory integration) and has a total NES score. We analysed subscale and total scores separately.

### Statistical Analyses

First, we used chi-square and t-tests for categorical and continuous variables respectively, to compare patients and controls. Next, Pearson’s correlation coefficient analyses were conducted to explore associations between childhood trauma and premorbid adjustment in the patient group. Finally, we assessed whether our selected risk variables played a moderating role in any associations between childhood trauma and premorbid adjustment. We used hierarchical regression analysis in cases where all of the variables were continuous. Regressions with and without interaction effects (between independent and moderating variables) were fitted and the F-to-remove test used to determine if the interaction effects made significant contributions to the R^2^. Significant contributions of the interaction terms were used as an indication of moderating effects. For the categorical moderator variables we performed an analysis of covariance for homogeneity of slopes. If the homogeneity of slopes tests (interaction between continuous independent and categorical moderator variables) were significant, this was taken to be an indication of moderating effects as it implies that correlations between independent and dependent variables are different for different levels of the moderator. Moderator variables that were considered were family history of schizophrenia, family history of psychiatric disorder, obstetric complications, neurological soft signs, and substance abuse. We used STATISTICA 13 software and, except where noted differently, statistical significance was set at p<0.05. Assumptions for the abovementioned tests were monitored throughout statistical procedures and were not found to be violated. Pearson correlations were augmented by calculating non-parametric Spearman correlations, which in most instances gave similar results. We, therefore, only report on Pearson correlations.

## Results

### Demographic and Clinical Characteristics and History of Childhood Trauma

The sample comprised 77 patients with schizophrenia (n = 52, 67.5%), schizophreniform disorder (n = 24, 31.2%) and schizoaffective disorder (n = 1, 1.3%), with a mean duration of untreated psychosis (DUP) of 32 weeks (SD = 34 weeks). DUP is defined as the number of weeks from the first onset of obvious psychotic symptoms to the first time antipsychotic treatment was started. There were 52 matched healthy controls. Patients and controls were similar in terms of demographic characteristics and CTQ scores (Table 1). We also categorized participants according to their exposure to high or low levels of childhood trauma, using previously described cut-off scores [22]. According to this categorisation a score of 5–12 for emotional abuse is regarded as low and 13–25 as high. For physical abuse and physical neglect a score of 5–9 is categorized as low and 10–25 as high;, for sexual abuse a score of 5–7 is low and 8–25 is high; and for emotional neglect a score of 5–14 is categorized as low and 15–25 as high. Exposure to high levels of trauma was reported for patients and controls respectively, as follows: emotional abuse 22(29%) and 13(25%) (χ^2^ 0.29, p = 0.6); physical abuse 29(39%) and 15(29%) (χ^2^ 1.43, p = 0.2); sexual abuse 22(29%) (χ^2^ 0.09, p = 0.8) and 14(27%); emotional neglect 23(31%) and 14(27%) (χ^2^0.2, p = 0.6); physical neglect 33(43%) and 22(42%) (χ^2^ 0.04, p = 0.9); and total trauma score 23(30%) and 13(25%) (χ^2^0.36, p = 0.5).

**Table 1:** Demographic details and childhood trauma scores for patients and controls.

		Patients	Controls	P*
		(n = 77)	(n = 52)	
Age yrs, mean(SD)		24.7(7.2)	25.1(6.8)	0.7
Males, n(%)		56(72.7%)	35(67.3%)	0.5
Highest level of education, n(%)				0.6
	Primary	4(3%)	3(2%)	
	Secondary	48(37%)	25(19%)	
	Grade 12 completed	19(15%)	19(15%)	
	Tertiary	4(3%)	4(3%)	
	Technical	2(2%)	1(1%)	
Ethnicity, n(%)				0.3
	Black	10(13%)	9(17.3%)	
	Mixed Ethnicity	62(80.5%)	36(69.2%)	
	White	5(6.5%)	7(13.5%)	
CTQ Scores, mean(SD)				
	EA	10.1(5.2)	9.8(4.6)	0.7
	PA	9.8(5.3)	8.8(4.4)	0.3
	SA	7.8(4.9)	7.6(4.5)	0.8
	PN	9.3(3.5)	9(3.6)	0.7
	EN	11.8(5.5)	11(5.6)	0.4
	Overall	47.6(17.1)	46.2(15.6)	0.6

* T-tests for continuous variables and Chi square test for categorical variables

CTQ = Childhood Trauma Questionnaire; EA = Emotional Abuse Subscale; PA = Physical Abuse Subscale; SA = Sexual Abuse Subscale; PN = Physical Neglect Subscale; EN = Emotional Neglect Subscale.

### Correlations between Childhood Trauma and Premorbid Adjustment Scores

There were several statistically significant correlations between CTQ and PAS scores, although the effect sizes were in the small (r = 0.1–0.3) to medium (r = 0.3–0.5) range (Table 2). Significant correlations were apparent across all of the CTQ categories, and in both social and academic domains across all of the PAS life stages.

**Table 2:** Pearson correlations between childhood trauma and premorbid adjustment scores for the 77 patients.

CTQ	PAS Overall	PAS Domain	PAS Childhood	PAS Early Adolescence	PAS Late Adolescence
EA	0.29*	Social	0.08	0.23	0.23
		Academic	0.27*	0.20	0.11
PA	0.32**	Social	0.10	0.15	0.21
		Academic	0.30**	0.26*	0.25*
SA	0.38***	Social	0.17	0.28*	0.35**
		Academic	0.27*	0.22	0.19
PN	0.37***	Social	0.16	0.29*	0.20
		Academic	0.31**	0.36**	0.23
EN	0.15	Social	-0.13	-0.02	0.10
		Academic	0.11	0.27*	0.26*
Overall	0.38***	Social	0.08	0.24*	0.29*
		Academic	0.34**	0.32**	0.26*

*p≤.05;

**p≤.01;

***p≤.001

PAS = Premorbid Adjustment Scale; CTQ = Childhood Trauma Questionnaire; EA = Emotional Abuse Subscale; PA = Physical Abuse Subscale; SA = Sexual Abuse Subscale; PN = Physical Neglect Subscale; EN = Emotional Neglect Subscale.

### Interactions between Moderators and Childhood Trauma

**Fig 1:**
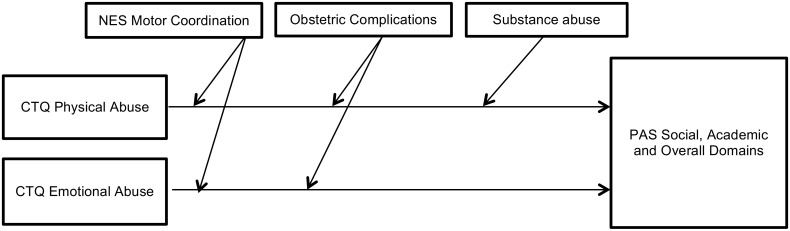
Moderator variables weakening the association between childhood trauma and premorbid adjustment.

**Fig 2:**
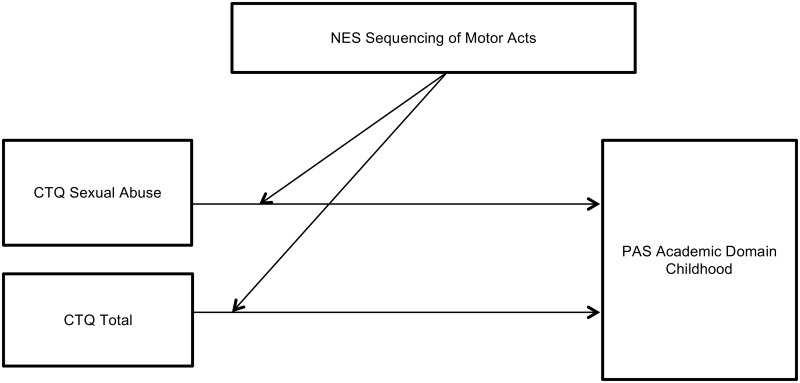
Moderator variable strengthening the association between childhood trauma and premorbid adjustment.

Statistically significant interactions that moderate the strength of the associations between childhood trauma and premorbid adjustment are presented in Tables 3 and 4. We found no moderating effects for a family history of schizophrenia or a family history of psychiatric illness on the association between childhood trauma and premorbid adjustment. However, other variables had significant moderating effects—either weakening (Fig 1) or strengthening (Fig 2)–the association between several types of childhood trauma and both social and academic premorbid adjustment from childhood to late adolescence. Poor motor coordination weakened the association between childhood emotional abuse and premorbid adjustment for the social, academic and overall domains through all the life stages except for the social domain in late adolescence. Poor motor coordination also weakened the association between childhood physical abuse and social adjustment from childhood to late adolescence and the PAS overall score. A history of obstetric complications weakened the association between childhood physical and emotional abuse and CTQ total score on social adjustment in childhood. It also weakened the association between emotional abuse and academic adjustment in childhood and the PAS overall score. The presence of substance abuse weakened the association between physical abuse and social adjustment in late adolescence. Moderator variables that strengthened the association between childhood trauma and premorbid adjustment were as follows: poor motor sequencing performance strengthened the relationship between sexual abuse and academic adjustment in childhood; and poor motor sequencing performance also strengthened the association between CTQ total score and academic adjustment in childhood.

**Table 3:** Continuous variables: moderator effects on the association between childhood trauma and premorbid adjustment.

Moderator	CTQ	PAS	Interaction Effect	
			R^2 Change	*p*
NES Motor Coordination	Emotional Abuse	Academic Domain Childhood	-0.12	<0.01
		Social Domain Early Adolescence	-0.06	0.04
		Academic Domain Early Adolescence	-0.10	0.01
		Academic Domain Late Adolescence	-0.08	0.02
		Overall	-0.15	0.00
	Physical Abuse	Social Domain Early Adolescence	-0.10	0.01
		Social Domain Late Adolescence	-0.06	0.05
		Overall	-0.08	0.01
NES Motor Sequencing	Sexual Abuse	Academic Domain Childhood	-0.07	0.02
NES Total	Physical Neglect	Academic Domain Childhood	-0.06	0.03

PAS = Premorbid Adjustment Scale; CTQ = Childhood Trauma Questionnaire; NES = Neurological Evaluation Scale.

**Table 4:** Categorical variables: moderator effects on the association between childhood trauma and premorbid adjustment.

Moderator		CTQ	PAS	Interaction Effect		Individual Effect	
				F	P	F	*p*
Obstetric Complications	Present	Physical Abuse	Social Domain Childhood	5.0	0.3	0.49	0.04
	Absent	Physical Abuse	Social Domain Childhood			-0.08	0.6
	Present	Emotional Abuse	Social Domain Childhood	6.20	0.02	0.50	0.02
	Absent	Emotional Abuse	Social Domain Childhood			-0.09	0.5
	Present	Emotional Abuse	Academic Domain Childhood	4.86	0.03	0.58	0.01
	Absent	Emotional Abuse	Academic Domain Childhood			0.16	0.3
	Present	Emotional Abuse	Overall	4.13	0.05	0.65	0.00
	Absent	Emotional Abuse	Overall			0.17	0.2
	Present	Overall	Social Domain Childhood	8.53	0.01	0.56	0.01
	Absent	Overall	Social Domain Childhood			-0.13	0.4
Substance Abuse	Present	Physical Abuse	Social Domain Late Adolescence	6.21	0.02	-0.10	0.6
	Absent	Physical Abuse	Social Domain Late Adolescence			0.47	0.00

PAS = Premorbid Adjustment Scale; CTQ = CTQ = Childhood Trauma Questionnaire.

## Discussion

In contrast to several previous studies [26,15, 17, 27–28] we did not find higher rates of childhood trauma in patients compared with matched controls. However, this may be explained by the fact that the rates of childhood trauma documented in both patients and controls in this study were generally higher than those reported in other studies. For example, in our sample 29.3% patients and 26.9% controls endorsed high levels of sexual abuse and 39.2% patients and 28.8% controls endorsed high levels physical abuse. In comparison, in a UK-based study, 16% of first episode psychosis patients and 13% controls reported severe sexual abuse and 24% patients and 13% controls reported severe physical abuse [26]. In a study conducted in Switzerland, 12% patients reported sexual abuse and 17.7% reported physical abuse [17]. Our findings are consistent with those of a meta-analysis that compared rates of childhood trauma across countries and found that that Lower and Middle Income Countries (LMIC) had higher rates than upper income countries [29]. Notably, LMICs have higher rates of crime, poverty and unemployment—all of which are socio-economic factors known to influence childhood trauma rates [30–31]. Alternatively, the high rates of reported childhood trauma in our sample may relate to measurement with the CTQ. Although the CTQ is extensively used it is a subjective self-report instrument and unlike clinician-rated instruments more likely to lead to over-reporting. Our finding that physical neglect was the most commonly cited childhood trauma type in both patients and controls is consistent with other studies [15, 32]. Tikka [15] found this to be the case in both ‘at clinical high risk patients’ and controls, while Wang [32] found this for patients with both first-episode and chronic schizophrenia. However, another study found that emotional and not physical neglect was most frequently reported in patients with first-episode psychosis [12].

Our study adds to the evidence for an association between exposure to childhood maltreatment and poor premorbid adjustment in schizophrenia spectrum disorder [14–17]. While an association does not imply causality, one explanation for these findings is that childhood adversity in some way contributes to impaired neurodevelopment, which manifests prior to the onset of psychosis as poor premorbid adjustment. The present study extends prior research in several important ways. First, we found that the relationships between childhood trauma and premorbid adjustment were not-specific—they spanned all forms of trauma, i.e. emotional and physical abuse and neglect and sexual abuse. Childhood trauma correlated with both academic and social adjustment, and across all of the life stages. Further evidence for a general rather than specific association derives from the finding of a moderate effect-size correlation between total trauma scores and overall premorbid adjustment. Also, all CTQ subscales other than emotional neglect correlated significantly with the overall PAS score. However, while the relationships were significant, they were not strong, with effect sizes in the small to medium range. This suggests that, in addition to childhood trauma, other factors contribute to poor premorbid adjustment in psychosis.

The association that we found between physical abuse and academic adjustment from childhood through to late adolescence is consistent with work indicating that physical abuse has a particular impact on academic achievement [33]. These authors compared children with and without exposure to trauma in terms of academic achievement and found that those who had experienced physical abuse had poorer academic achievement. Our findings also mirror those of Alameda [17] in so far as we also found an association between sexual abuse and poor social adjustment in adolescence. Alameda [17] found that sexual abuse correlated significantly with poor social functioning in early adolescence, while in our study we found sexual abuse correlated with social functioning in both early and late adolescence. However, unlike the Alameda [17] study we did not find an association between physical abuse and premorbid social adjustment.

While there has been considerable interest in the role of sexual and physical abuse in psychosis, there has been less focus on emotional abuse and physical and emotional neglect [34]. In the current study, physical neglect was significantly correlated with poorer academic adjustment during childhood and early adolescence and with poorer social adjustment in early adolescence. The physical neglect subscale includes items relating to the provision of basic physical needs such as food and shelter. Poor diet is known to affect academic performance in childhood. Unlike older adolescents, who are likely to be more advanced in terms of their functional development, children and young adolescents may be less able to fend for themselves and seek proper food and shelter and thus more vulnerable to physical neglect. In our study, emotional neglect was significantly associated with academic adjustment throughout adolescence, but not with social adjustment. This could perhaps reflect the important parental role in academic performance during adolescence, whereas peer support may compensate to some degree for social adjustment needs. Also we found that emotional abuse was significantly associated with academic adjustment in childhood. Emotionally abused and neglected children and adolescents have been shown to have more severe cognitive and academic deficits as evidenced by lower general intelligence, poorer numeracy and literacy skills, and more frequent repetition of grades at school [35]. One possible explanation is that emotional abuse may be associated with dissociation [36]. A recent study suggests that children with high rates of dissociative symptoms are more likely to have poor academic functioning, due to their impact on memory, learning and engagement in classroom activities [37]. Early maltreatment has been linked to dissociative symptoms in patients with psychosis, with emotional abuse showing a particularly robust association [38].

Contrary to expectation, the results of our moderator analyses did not provide support for a model incorporating a cumulative effect of risk factors in schizophrenia, at least not in a directional pathway that includes childhood trauma. Thus, we did not find, as hypothesized, that genetic predisposition strengthened the association between childhood trauma and premorbid adjustment. While a family history of schizophrenia or psychiatric disorder may not be the ideal manner of assessing genetic risk, it has in fact been argued that it does capture a greater proportion of genetic load, compared with direct molecular genetic measurement [39]. Indeed, using this method, Trotta [39] explored the impact of the interplay between childhood adversity and family psychiatric history on the onset of psychosis and found no evidence that childhood adversity and familial liability combined synergistically to increase the odds of psychosis beyond the effect of each individually. This is consistent with our findings, insofar as our results do not support a role for familial liability in amplifying the effect of childhood adversity on premorbid adjustment. Furthermore, family history of a psychiatric illness or schizophrenia did not have a significant interaction effect on the relationship between childhood trauma and premorbid adjustment. This is also consistent with the findings of Trauelsen [27] who reported that family history of psychiatric disease was not associated with deteriorating premorbid adjustment.

Further, the two environmental risk factors whose interactive effect we assessed actually weakened the strength of some of the associations between childhood trauma and premorbid adjustment. A history of obstetric complications significantly weakened the associations between childhood physical abuse, emotional abuse and CTQ total score and social adjustment in childhood. It also weakened the associations between emotional abuse and both academic adjustment in childhood and PAS overall score. Substance abuse significantly reduced the strength of the association between physical abuse and social adjustment in late adolescence. The findings of an inverse moderating effect for obstetric complications and substance abuse on the interaction between childhood trauma and premorbid adjustment suggest the existence of different, and at least partly independent, pathways to psychosis. It may be that the “affective pathway to psychosis”, in which childhood trauma is proposed to play a role [40], is relatively independent of the effects of genetic loading, and particularly obstetric complications and substance abuse. Indeed, the negative interaction effects of obstetric complications and substance abuse suggest that these environmental risk factors operate via an alternative mechanism, not involving childhood trauma.

Our findings are also consistent with the observation that, in studies investigating childhood trauma in psychosis that have controlled for factors such as cannabis use and family history of psychosis, the effect of childhood trauma remained significant, suggesting that childhood trauma as a risk factor is at least partially independent of these other factors [41]. However, contrary to our finding of a negative interaction effect of substance abuse on the association between childhood trauma and premorbid adjustment, others have found that cannabis use and childhood trauma interact additively to increase the risk of psychotic symptoms in adolescence [42], and individuals with a history of non-consensual sexual experience and cannabis consumption are over seven times more likely to have been diagnosed with psychosis compared with those without these experiences [43].

The moderating effect of neurological soft signs on the relationship between childhood trauma and premorbid adjustment is of interest. Our results indicate that in patients with poor motor coordination skills, the relationship between childhood trauma and poor premorbid adjustment is weaker. Specifically, poor motor coordination weakened the effect of emotional abuse on social and academic premorbid adjustment through all of the life stages, except the social domain in late adolescence, and also had this effect on overall PAS score. It also weakened the association between childhood physical abuse and social adjustment from childhood to late adolescence. On the other hand, poor motor sequencing performance strengthened the relationship between sexual abuse and academic adjustment in childhood as well as the association between CTQ total score and academic adjustment in childhood. Assuming that neurological soft signs represent clinical manifestations of neurodevelopmental compromise [44], it may be that neurological soft signs could be useful biological markers for identifying different pathways to psychosis. Thus, individuals whose neurodevelopmental compromise manifests as poor motor sequencing skills may represent those in whom childhood trauma is important aetiologically. Conversely, individuals with neurodevelopmental compromise that manifests as poor motor coordination may represent those where other risk factors, such as obstetric complications and substance abuse play an important role.

There are several limitations to this study that need to be considered:

- A larger sample would have provided more power to detect additional interactions.
- This paper is exploratory of nature, and we did not conduct power calculations.
- As this was a cross-sectional study we are not able to make inferences about causality.
- As with all assessments of childhood trauma exposure, we relied on retrospective recall which may introduce bias. However, it has been reported that memory bias explains little of the variance in assessments of childhood trauma [45].
- Family history of schizophrenia and psychiatric disorder may not adequately represent genetic vulnerability for schizophrenia, despite the argument that it captures a greater portion of genetic load than molecular genetic measurements [39].
- Although patients and controls were recruited from the same geographical catchment area, known for high levels of poverty and carefully matched in terms education, ethnicity, gender and age, we did not collect information on socioeconomic status. Differences in socioeconomic status may explain why we did not find any significant differences in the distribution of different trauma types across patients and controls.
- We did not use a standardized psychometric instrument to assess substance use. Information on substance use was gathered from clinical interviews with patients and through collateral from caregivers, and through urine drug tests.

## Conclusion

In conclusion, our results confirm previous studies indicating an association between childhood trauma and premorbid adjustment, and suggest that this association is apparent for different types of trauma, and for both social and academic components of premorbid adjustment across childhood, early and late adolescence. Further, our results suggest a complex interplay of various risk factors, supporting the notion of different pathways to psychosis. Our results suggesting that childhood trauma might act largely independently of pre-existing genetic liability, obstetric complications and substance abuse count against a simple cumulative model of the shared effect of adversities on the risk of psychosis. It may be that individuals with psychosis have different adversity profiles [27]. Further studies exploring the ways in which putative risk factors for neurodevelopmental compromise and the evolution of psychosis interact with one another are warranted.

## References

[1] Murray RM, McDonald C, Bramon E. Neurodevelopmental impairment, dopamine sensitisation, and social adversity in schizophrenia. World Psychiatry. 2002; 1(3):137–145. 16946834 PMC1489849

[2] Cannon-Spoor HE, Potkin SG, Wyatt RJ. Measurement of premorbid adjustment in chronic schizophrenia. Schizophr Bull. 1982; 8(3):470–84. 7134891 10.1093/schbul/8.3.470

[3] Tarbox SI, Brown LH, Haas GL. Diagnostic specificity of poor premorbid adjustment: comparison of schizophrenia, schizoaffective disorder, and mood disorder with psychotic features. Schizophr Res. 2012; 141(1):91–7. 10.1016/j.schres.2012.07.008 22858353 PMC3438358

[4] Murray RM, Jones P, O'Callaghan E, Takei N, Sham P. Genes, viruses and neurodevelopmental schizophrenia. J Psychiatr Res. 1992; (4):225–35. 10.1016/0022-3956(92)90029-n 1491349

[5] Gupta SKP. What is schizophrenia: A neurodevelopmental or neurodegenerative disorder or a combination of both? A critical analysis. Indian J Psychiatry. 2010; 52(1):21–27. 10.4103/0019-5545.58891 20174514 PMC2824976

[6] Petruzzelli MG, Margari L, Craig F, Campa MG, Martinelli D, Pastore A, et al Markers of neurodevelopmental impairments in early-onset psychosis. Neuropsychiatr Dis Treat. 2015; 11:1793–1798. 10.2147/NDT.S83904 26229474 PMC4516349

[7] Varese F, Smeets F, Drukker M, Lieverse R, Lataster T, Viechtbauer W, et al Childhood Adversities Increase the Risk of Psychosis: A Meta-analysis of Patient-Control, Prospective- and Cross-sectional Cohort Studies. Schizophr Bull. 2012; 38(4):661–671. 10.1093/schbul/sbs050 22461484 PMC3406538

[8] Schafer I, Harfst T, Aderhold V, Briken P, Lehmann M, Moritz S, et al Childhood trauma and dissociation in female patients with schizophrenia spectrum disorders an exploratory study. Nerv Ment Dis. 2006; 194:135–138. 10.1097/01.nmd.0000198199.57512.84 16477194

[9] Glaser JP, Van Os J, Portegijs PJM, Myin-Germeys I. Childhood trauma and emotional reactivity to daily life stress in adult frequent attenders of general practitioners. J Psychosom Res. 2006; 61(2):229–236. 10.1016/j.jpsychores.2006.04.014 16880026

[10] Myin-Germeys I, Van Os J. Stress-reactivity in psychosis: Evidence for an affective pathway to psychosis. Clin Psychol Rev. 2007; 27:409–424. 10.1016/j.cpr.2006.09.005 17222489

[11] Krabbendam L. Childhood psychological trauma and psychosis. Psychol Med. 2008; 38:1405–1408. 10.1017/S0033291708002705 18257939

[12] Ücok A, Bikmaz S. The effects of childhood trauma in patients with first-episode schizophrenia. Acta Psychiatr Scand. 2007; 116:371–377. 10.1111/j.1600-0447.2007.01079.x 17919156

[13] Thompson AD, Nelson B, Yuen HP, Lin A, Amminger GP, McGorry PD, et al (2014). Sexual trauma increases the risk of developing psychosis in an ultra high-risk “Prodromal” population. Schizophr Bull. 2014; 40(3):697–706. 10.1093/schbul/sbt032 23455040 PMC3984502

[14] Conus P, Ward J, Lucas N, Cotton S, Yung AR, Berk M, et al Characterisation of the prodrome to a first episode of psychotic mania: results of a retrospective study. J Affect Disord. 2010; 124:341–345. 10.1016/j.jad.2009.12.021 20085850

[15] Tikka M, Luutonen S, Ilonen T, Tuominen L, Kotimäki M, Hankala J, et al Childhood trauma and premorbid adjustment among individuals at clinical high risk for psychosis and normal control subjects. Early Interv Psychiatry. 2013; 7:51–57. 10.1111/j.1751-7893.2012.00391.x 22925391

[16] Stain HJ, Brønnick K, Hegelstad WTV, Joa I, Johannessen JO, Langeveld J, et al Impact of interpersonal trauma on the social functioning of adults with first-episode psychosis. Schizophr Bull. 2014; 40(6):1491–1498. 10.1093/schbul/sbt166 24282322 PMC4193690

[17] Alameda L, Ferrari C, Baumann PS, Gholam-Rezaee M, Do KQ, Conus P. Childhood sexual and physical abuse: Age at exposure modulates impact on functional outcome in early psychosis patients. Psychol Med. 2015; 45:2727–2736. 10.1017/S0033291715000690 26350397

[18] Demjaha A, MacCabe JH, Murray RM. How genes and environmental factors determine the different neurodevelopmental trajectories of schizophrenia and bipolar disorder. Schizophr Bull. 2012; 38(2):209–14. 10.1093/schbul/sbr100 21857009 PMC3283142

[19] Quee PJ, Meijer LJH, Islam A, Aleman A, Alizadeh BZ, Meijer CJ, et al Premorbid adjustment profiles in psychosis and the role of familial factors. J Abnorm Psychol. 2014; 123(3):578–587. 10.1037/a0037189 25000154

[20] First MB, Spitzer RL, Gibbon M, Williams JBW. Structured Clinical Interview for DSM-IV-TR Axis I Disorders, Research Version, Non-patient Edition. (SCID-I/NP) New York: Biometrics Research, New York State Psychiatric Institute, 11 2002.

[21] Bernstein DP, Stein JA, Newcomb MD, Walker E, Pogge D, Ahluvalia T, et al Development and validation of a brief screening version of the childhood trauma questionnaire. Child Abuse Negl. 2003; 27:169–190. 12615092 10.1016/s0145-2134(02)00541-0

[22] Bernstein DP, Fink LA. CTQ: Childhood trauma questionnaire: A retrospective self-report. 1998 Harcourt Brace & Company, San Antonio.

[23] Norman RMG, Malla AK, Manchanda R, Townsend L. Premorbid adjustment in first episode schizophrenia and schizoaffective disorders: a comparison of social and academic domains. Acta Psychiatr Scand. 2005; 112:30–39. 10.1111/j.1600-0447.2005.00555.x 15952943

[24] Allen DN, Strauss GP, Barchard KA, Vertinski M, Carpenter WT, Buchanan RW. Differences in developmental changes in academic and social premorbid adjustment between males and females with schizophrenia. Schizophr Res. 2013; 146:132–137. 10.1016/j.schres.2013.01.032 23490759 PMC3752849

[25] Buchanan RQ, Heinrichs DW. The neurological evaluation scale (NES): A structured instrument for the assessment of neurological signs in schizophrenia. Psychiatry Res. 1998; 21:335–350. 10.1016/0165-1781(89)90148-0 2710870

[26] Aas M, Dazzan P, Fisher HL, Morgan C, Morgan K, Reichenberg A, et al Childhood trauma and cognitive function in first-episode affective and non-affective psychosis. Schizophr Res. 2011; 129:12–19. 10.1016/j.schres.2011.03.017 21601792

[27] Trauelsen A. M., Bendall S., Jansen J. E., Nielsen H. G., Pedersen M. B., Trier C. H., et al (2016). Childhood adversities: Social support, premorbid functioning and social outcome in first-episode psychosis and a matched case-control group. Aust N Z J Psychiatry. 2016;1–13. 10.1177/0004867415625814 26773690

[28] Sideli L, Fisher HL, Murray RM, Sallis H, Russo M, et al (2015). Interaction between cannabis consumption and childhood abuse in psychotic disorders: preliminary findings on the role of different patterns of cannabis use. Early Interv Psychiatry. 2015 11 12. 10.1111/eip.12285 26560802

[29] Viola TW, Salum GA, Kluwe-Schiavona B, Sanvicente-Vieiraa B, Levandowski ML, Grassi-Oliveira R. (2016). The influence of geographical and economic factors in estimates of childhood abuse and neglect using the childhood trauma questionnaire: A worldwide meta-regression analysis. Child Abuse Negl. 2016; 51:1–16. 10.1016/j.chiabu.2015.11.019 26704298

[30] Lowe SR, Quinn JW, Richard CA, Pothen J, Rundle A, Galeae S, et al Childhood trauma and neighborhood-level crime interact in predicting adult posttraumatic stress and major depression symptoms. Child Abuse Negl. 2016; 51:212–222. 10.1016/j.chiabu.2015.10.007 26499372 PMC4713249

[31] Ramsay CE, Flanagan P, Gantt S, Broussard B, Compton MT. Clinical correlates of maltreatment and traumatic experiences in childhood and adolescence among predominantly African-American, socially disadvantaged, hospitalized, first-episode psychosis patients. Psychiatry Res. 2011; 188:343–349. 10.1016/j.psychres.2011.05.019 21665293 PMC3146577

[32] Wang Z, Xue Z, Pu W, Yang B, Li L, Yi W, et al Comparison of first-episode and chronic patients diagnosed with schizophrenia: Symptoms and childhood trauma. Early Interv Psychiatry. 2013; 7:23–30. 10.1111/j.1751-7893.2012.00387.x 22947390

[33] Jonson-Reid M, Drake B, Kim J, Porterfield S, Han L. A prospective analysis of the relationship between reported child maltreatment and special education eligibility among poor children. Child Maltreatment. 2004; 9(4):382–394. 10.1177/1077559504269192 15538037

[34] Schenkel LS, Spaulding WD, DiLillo D, Silverstein SM. Histories of childhood maltreatment in schizophrenia: relationships with premorbid functioning, symptomatology, and cognitive deficits. Schizophr Res. 2005; 76:273–286. 10.1016/j.schres.2005.03.003 15949659

[35] Maguire SA, Williams B, Naughton AM, Cowley LE, Tempest V, Mann MK, et al A systematic review of the emotional, behavioural and cognitive features exhibited by school-aged children experiencing neglect or emotional abuse. Child: care, health and development. 2015; 41(5):641–653. 10.1111/cch.12227 25733080

[36] Sanders B, Giolas MH. Dissociation and childhood trauma in psychologically disturbed adolescents. Am J Psychiatry. 1991; 149(1):50–54. 10.1176/ajp.148.3.A50 1984706

[37] Perzow SED, Petrenko CLM, Garrido EF, Combs MD, Culhane SE, Taussig HN. Dissociative symptoms and academic functioning in maltreated children: A preliminary study. J Trauma Dissociation. 2013; 14(3):302–311. 10.1080/15299732.2012.736928 23627479 PMC4305440

[38] Braehler C, Valiquette L, Holowka D, Malla AK, Joober R, Ciampi A, et al Childhood trauma and dissociation in first-episode psychosis, chronic schizophrenia and community controls. Psychiatry Res. 2013; 210(1):36–42. 10.1016/j.psychres.2013.05.033 23816517

[39] Trotta A, Di Forti M, Iyegbe C, Green P, Dazzan P, Mondelli V, et al Familial risk and childhood adversity interplay in the onset of psychosis. Br J Psychiatry. 2015;1(1):6–13. 10.1192/bjpo.bp.115.000158 PMC4995579 27703716

[40] Van Winkel R, Stefanis NC, Myin-Germeys I. Psychosocial Stress and Psychosis. A Review of the Neurobiological Mechanisms and the Evidence for Gene-Stress Interaction. Schizophr Bull. 2008; 34(6):1095–1105. 10.1093/schbul/sbn101 18718885 PMC2632486

[41] Bendall S, Alvarez-Jimenez M, Nelson B, McGorry PD. Childhood trauma and psychosis: new perspectives on aetiology and treatment. Early Interv Psychiatry. 2013; 7(1):1–4. 10.1111/eip.12008 23356889

[42] Harley M, Kelleher I, Clarke MC, Lynch F, Arseneault L. Cannabis use and childhood trauma interact additively to increase the risk of psychotic symptoms in adolescence. Psychol Med. 2010; 40:1627–1634. 10.1017/S0033291709991966 19995476

[43] Houston JE, Murphy J, Shevlin M, Adamson G. Cannabis use and psychosis: re-visiting the role of childhood trauma. Psychol Med. 2011; 41(11):1–10. 21557896 10.1017/S0033291711000559

[44] Varambally S, Venkatasubramanian G, Gangadhar BN. Neurological soft signs in schizophrenia—The past, the present and the future. Indian J Psychiatry. 2012; 54(1):73–80. 10.4103/0019-5545.94653 22556444 PMC3339227

[45] Fergusson DM, Horwood LJ, Boden JM. Structural equation modeling of repeated retrospective reports of childhood maltreatment. Int J Methods Psychiatr Res. 2011; 20(2):93–104. 10.1002/mpr.337 21495111 PMC6878375

